# Supplementary material for: Direct recruitment of Mis18 to interphase spindle pole bodies promotes CENP-A chromatin assembly
Source: Curr Biol. Author manuscript; Available in PMC 2026 Feb 23. (PMC7618776; doi:10.1016/j.cub.2023.08.063)
Supplement: Supplemental Data [file EMS212567-supplement-Supplemental_Data.pdf]

Supplemental Information

Table S1: Manually curated proteins and categories for annotation of IP/LFQ-MS plots, Related to Figures 1, 2, 5, S1, and S2 and Data S1-S3

Table S2: Strains used in this study, Related to STAR methods

Table S3: DNA primers used in this study, Related to STAR methods

Table S4: Plasmids used in this study, Related to STAR methods

Data S1: LFQ-MS results of Sad1-3V5 IPs in *wt*, *csi1*Δ, *lem2*Δ, and *csi1*Δ*lem2*Δ, Related to Figures 1 and S1 and Table S1

(A) Analysis of Sad1-3V5 wt vs. untagged

(B) Analysis of Sad1-3V5 wt vs. *csi1*Δ

(C) Analysis of Sad1-3V5 wt vs. *lem2*Δ

(D) Analysis of Sad1-3V5 wt vs. *csi1*Δ*lem2*Δ

Data S2: LFQ-MS results of Sad1-TEV-3V5 IPs with cleavage, Related to Figures 2 and S2 and Table S1

(A) Analysis of Sad1-3V5 vs. Sad1-TEV140-3V5 -TEV

(B) Analysis of Sad1-TEV140-3V5 +TEV vs. Sad1-TEV140-3V5 -TEV

(C) Analysis Sad1-TEV60-3V5 +TEV vs. Sad1-TEV140-3V5 +TEV

(D) Analysis of Sad1-TEV60-3V5 +TEV vs. Sad1-TEV60-3V5 -TEV

Data S3: LFQ-MS results of Sad1-4A-3V5 IPs, Related to Figure 5 and Table S1

(A) Analysis of Sad1-3V5 vs. Sad1-4A-3V5

(B) Analysis of Untagged vs. Sad1-3V5

A

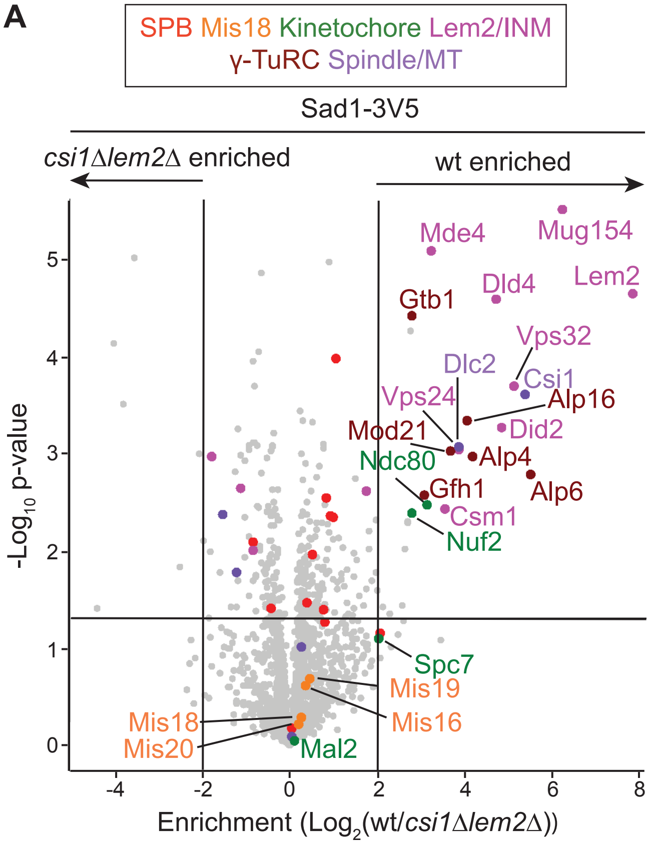

B

Sad1-3V5 enrichment

Genotype	Mis18C	Kinetochore
wt	+	+
csi1Δ	+	-
lem2Δ	+	+
csi1Δlem2Δ	+	-

Figure S1. IP/LFQ-MS of Sad1-3V5 in *csi1Δ lem2Δ* shows reduced association of similar proteins as individual *csi1Δ* and *lem2Δ* mutants combined. Related to Figure 1 and Data S1.

(A) Proteins detected in Sad1-3V5 IPs from wild-type (wt) and *csi1Δlem2Δ* cells were compared by LFQ-MS. Proteins with wt over *csi1Δlem2Δ* enrichments above $Log_2(2)$ and that were also depleted in either single mutant (Figure 1D-E) are highlighted and labelled in addition to Mis18 complex and kinetochore proteins.
(B) Table indicating whether Mis18 complex or kinetochore proteins are strongly copurified with Sad1-3V5 from cells with the indicated genotypes. Volcano plot protein category components are listed in Table S1 and color coded as indicated. See also Data S1A-S1D.

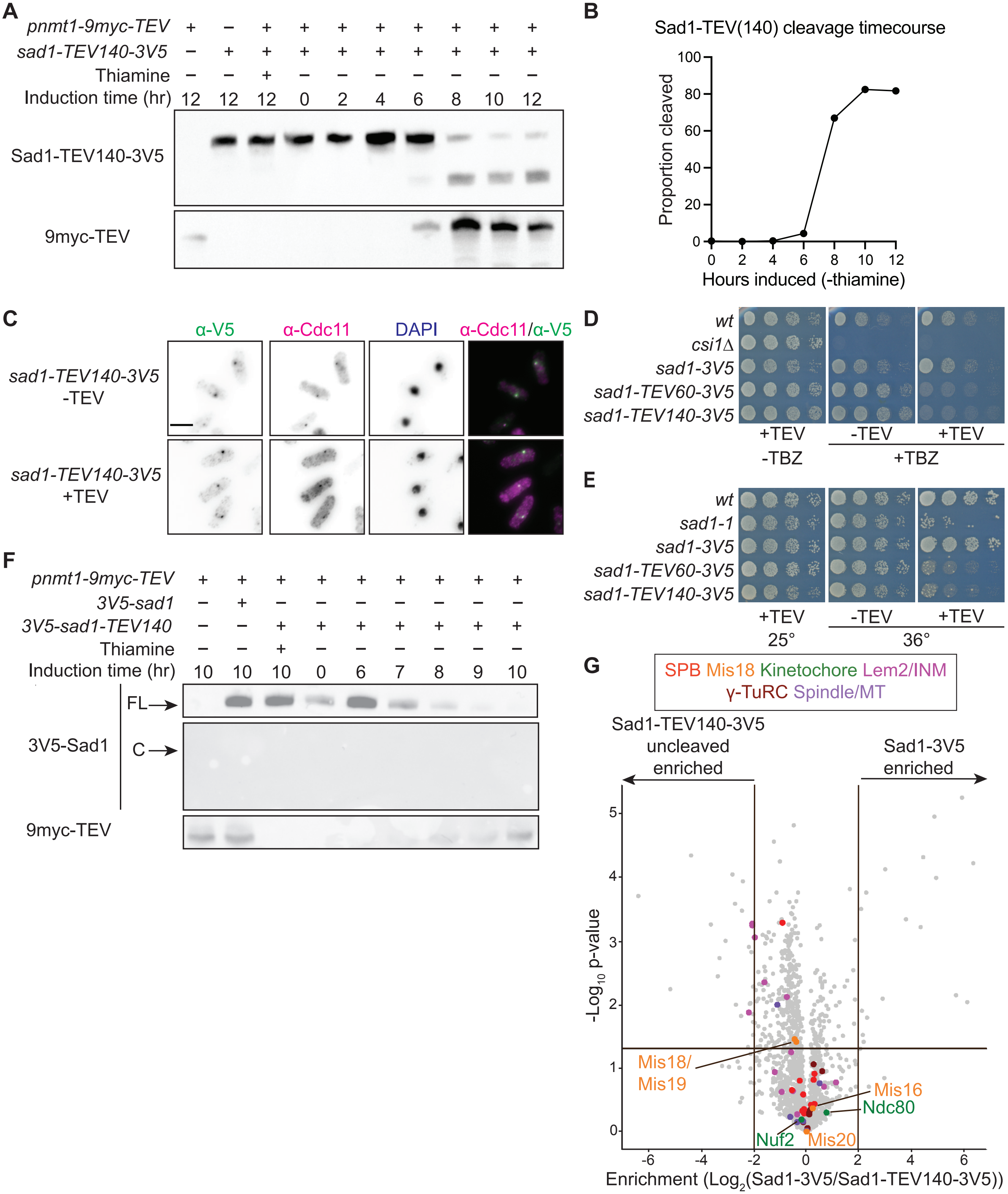
A
pnmt1-9myc-TEV
sad1-TEV140-3V5
Thiamine
Induction time (hr)
12 12 12 0 2 4 6 8 10 12
Sad1-TEV140-3V5
9myc-TEV
B
Sad1-TEV(140) cleavage timecourse
Proportion cleaved
Hours induced (-thiamine)
C
α-V5
α-Cdc11
DAPI
α-Cdc11/α-V5
sad1-TEV140-3V5 -TEV
sad1-TEV140-3V5 +TEV
D
wt
csi1Δ
sad1-3V5
sad1-TEV60-3V5
sad1-TEV140-3V5
+TEV
-TEV
+TEV
-TBZ
+TBZ
E
wt
sad1-1
sad1-3V5
sad1-TEV60-3V5
sad1-TEV140-3V5
+TEV
-TEV
+TEV
25°
36°
F
pnmt1-9myc-TEV
3V5-sad1
3V5-sad1-TEV140
Thiamine
Induction time (hr)
10 10 10 0 6 7 8 9 10
3V5-Sad1
FL
C
9myc-TEV
G
SPB Mis18 Kinetochore Lem2/INM
γ-TuRC Spindle/MT
Sad1-TEV140-3V5 uncleaved enriched
Sad1-3V5 enriched
-Log10 p-value
Mis18/ Mis19
Mis16
Ndc80
Nuf2
Mis20
Enrichment (Log2(Sad1-3V5/Sad1-TEV140-3V5))

Figure S2. Induced TEV cleavage of Sad1 nucleoplasmic region disrupts specific protein interactions, but not SPB localization of the remaining C-terminal portion of Sad1-TEV140-3V5. Related to Figure 2 and Data S2.

(A) Detection of Sad1-TEV140-3V5 cleavage (top) and TEV induction (bottom) during a time course by anti-V5 and anti-myc westerns, respectively. Cells from rich media (+thiamine) were washed and placed in +thiamine or -thiamine media at T=0.
(B) Quantification of Sad1-TEV140-3V5 cleavage levels from time course in A. Proportion cleaved = cleaved/(cleaved + uncleaved).
(C) Sad1-TEV140-3V5 immunolocalization relative to SPBs (anti-Cdc11) with (-) or without (+) TEV cleavage following TEV induction by thiamine removal for 12 hours. Scale bar: 5 μm.
(D) Serial dilution growth assays of *sad1-3V5, sad1-TEV60-3V5* and *sad1-TEV140-3V5* cells plated on defined media with (-TEV) or without (+TEV) thiamine that also did (+) or did not (-) include 12.5 μg/mL TBZ at 25 or 36°C. Wild-type (*wt*) and *csi1*Δ included as controls for TBZ sensitivity.
(E) Serial dilution growth assays of *sad1-3V5, sad1-TEV60-3V5* and *sad1-TEV140-3V5* cells plated on defined media with (-TEV) or without (+TEV) thiamine incubated at 25 or 36°C. Wild-type (*wt*) and *sad1-1* cells included as controls for temperature sensitivity.
(F) Probe for full length (FL) N-terminally tagged 3V5-Sad1-TEV140 (top) with TEV induction (bottom), and the expected N-terminal region (3V5-Sad1-140) cleaved (C) fragment (middle), with anti-V5 and anti-myc westerns respectively. Cells from rich media (+thiamine) were washed and placed in -thiamine media at T=0.
(G) Comparison of proteins enriched in Sad1-3V5 and uncleaved Sad1-TEV140-3V5 immunoprecipitates by IP/LFQ-MS. Volcano plot protein category components are listed in Table S1 and color coded as indicated. See also Data S2A.

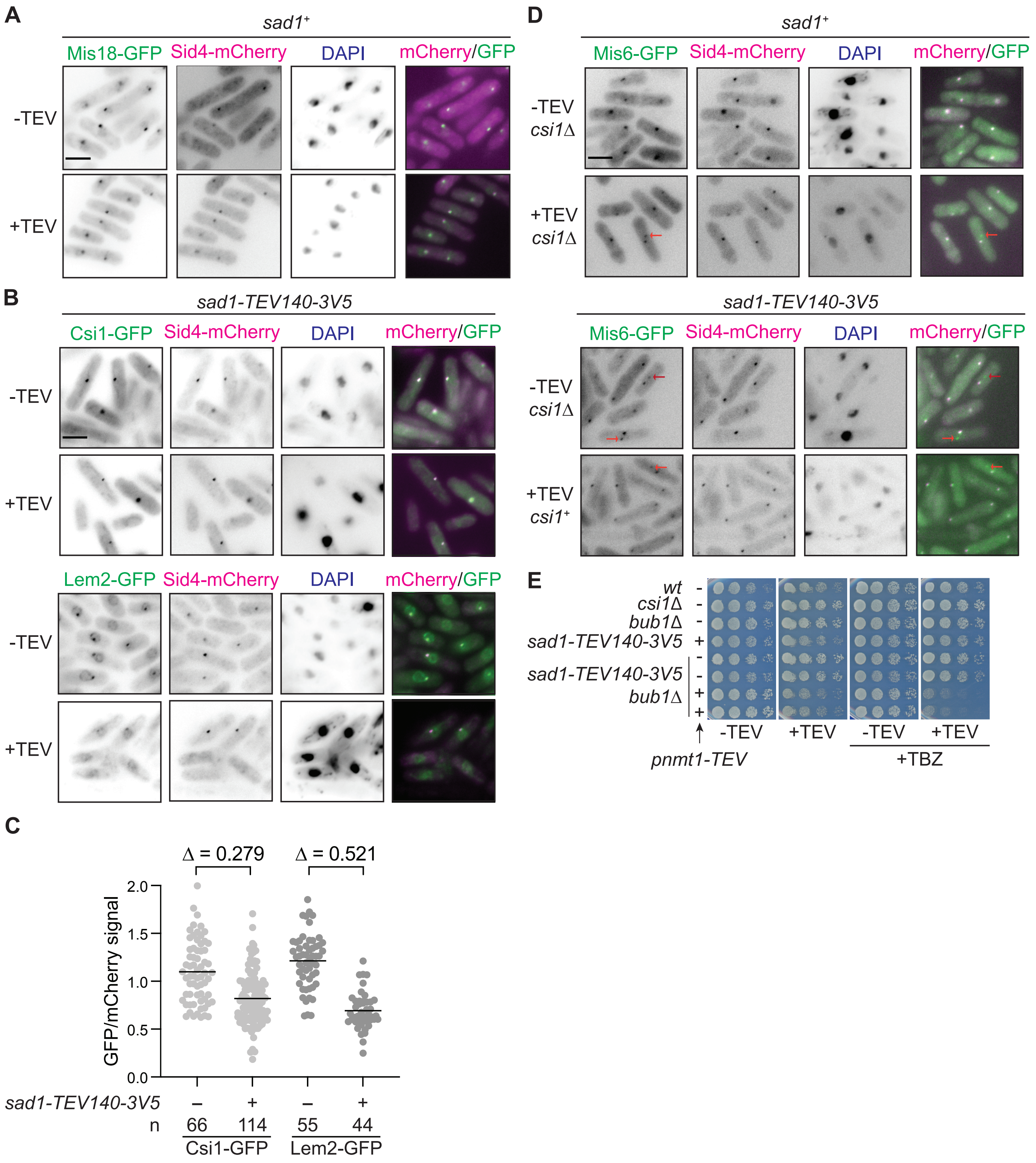

A
sad1+
Mis18-GFP
Sid4-mCherry
DAPI
mCherry/GFP
-TEV
+TEV
B
sad1-TEV140-3V5
Csi1-GFP
Sid4-mCherry
DAPI
mCherry/GFP
-TEV
+TEV
Lem2-GFP
Sid4-mCherry
DAPI
mCherry/GFP
-TEV
+TEV
C
Δ = 0.279
Δ = 0.521
GFP/mCherry signal
2.0
1.5
1.0
0.5
0.0
sad1-TEV140-3V5
–
+
–
+
n
66
114
55
44
Csi1-GFP
Lem2-GFP
D
sad1+
Mis6-GFP
Sid4-mCherry
DAPI
mCherry/GFP
-TEV csi1Δ
+TEV csi1Δ
sad1-TEV140-3V5
Mis6-GFP
Sid4-mCherry
DAPI
mCherry/GFP
-TEV csi1Δ
+TEV csi1+
E
wt -
csi1Δ -
bub1Δ -
sad1-TEV140-3V5 +
sad1-TEV140-3V5 bub1Δ - - + +
-TEV
+TEV
-TEV
+TEV
pnmt1-TEV
+TBZ

Figure S3. Cells with cleaved Sad1-TEV140-3V5 have aberrant Lem2 localization and are sensitive to Bub1 loss. Related to Figure 3.

(A) Representative images of Mis18-GFP and Sid4-mCherry in *sad1⁺* cells with (+TEV) or without (-TEV) TEV protease expression. Compare to *sad1-TEV140-3V5* cells in Figure 3A. Quantified in Figure 3B.
(B) Localization of Csi1-GFP (top) or Lem2-GFP (bottom) relative to SPBs (Sid4-mCherry) in *sad1-TEV140-3V5* cells with (+) or without (-) TEV expression for 16 hrs and stained with DAPI.
(C) Intensities of Csi1-GFP (light grey) and Lem2-GFP (dark grey) were measured in strains with or without Sad1-TEV140-3V5 cleavage and normalized to Sid4-mCherry signal. The difference in mean intensities are given as Δ. Scale bar: 5 μm.
(D) Representative images of Mis6-GFP and Sid4-mCherry localization in *sad1⁺* or *sad1-TEV140-3V5* cells with (+TEV) or without (-TEV) TEV protease expression. Csi1 is absent from *csi1*Δ cells. Compare to cells in Figure 3C. Quantified in Figures 3D and 3E. Arrows indicate Mis6-GFP separated from the SPB.
(E) Serial dilution growth assays on plates with (+) or without (-) TEV expression. *pnmt1-TE*V indicates presence (+) or absence (-) of an integrated allele of TEV protease under the *nmt1* promoter. TBZ was used at 7.5 μg/mL.

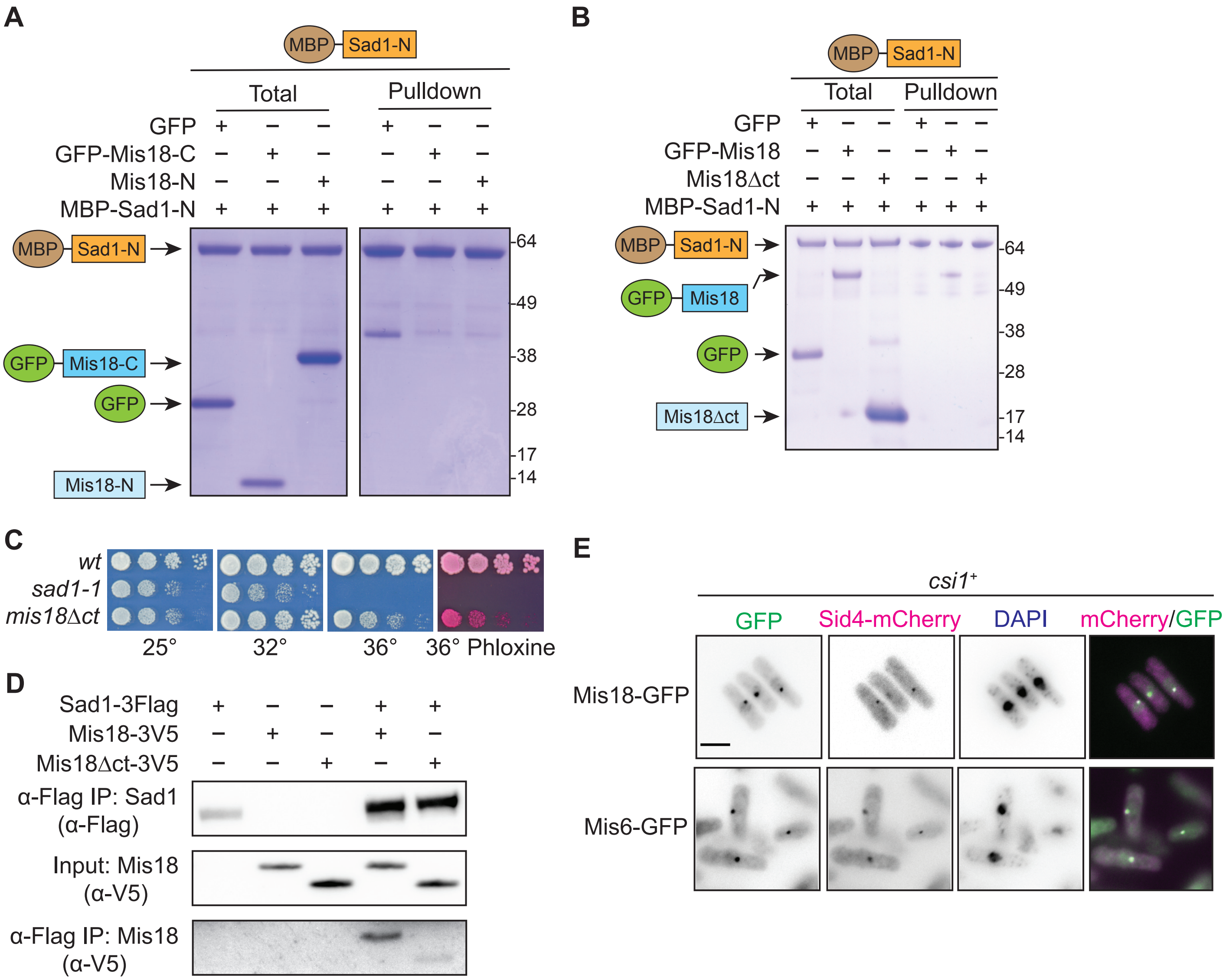

Figure S4. Mis18 truncations fail to bind the Sad1 nucleoplasmic region *in vitro.* Related to Figure 4.

(A) *In vitro* binding assays for recombinant N-terminal (residues 1-120) and C-terminal (residues 121-194, fused to GFP) portions of Mis18 or GFP (control) to MBP-Sad1-N (residues 2-167, nucleoplasmic region) bound to amylose resin. Size markers: kDa.
(B) *In vitro* binding assays for recombinant full length Mis18 fused to GFP, Mis18Δct (missing C-terminal residues 169-194) or GFP to MBP-Sad1-N (residues 2-167) bound to amylose resin.
(C) Serial dilution growth assays of wild-type (*wt*), *sad1-1* and *mis18Δct* cells on YES plates at indicated temperatures and in the presence of phloxine, where red staining indicates inviable cells.
(D) Western analyses of Input (extract) and anti-Flag Sad1-3Flag IPs to detect Sad1-3Flag (anti-Flag), Mis18-3V5 or Mis18Δct-3V5 (anti-V5).
(E) Localization of Mis18-GFP (top) or Mis6-GFP (bottom) and SPB protein Sid4-mCherry in *csi1*$^{+}$ DAPI stained cells. Compare to Figure 4D. Quantified in Figure 4E. Scale: 5 µm.

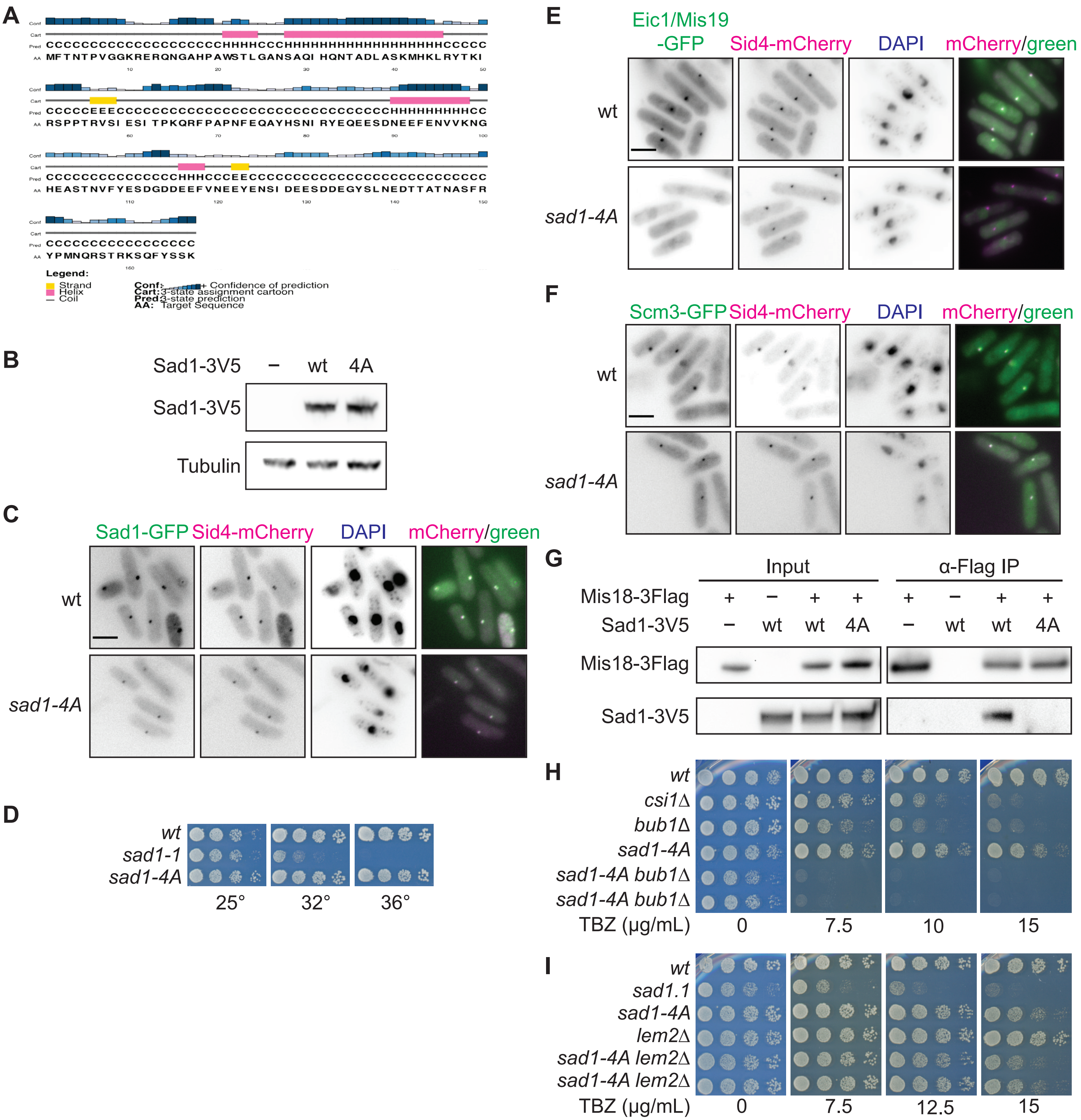

A
Conf
Cart
Pred CCCCCCCCCCCCCCCCCCCHHHHCCCHHHHHHHHHHHHHHHHHHHCCCCC
AA MFTNTPVGGKRERQNGAHPAWSTLGANSAQIHQNTADLASKMHKLRYTKI
10 20 30 40 50
Pred CCCCCEEECCCCCCCCCCCCCCCCCCCCCCCCCCCCCHHHHHHHHHHHCC
AA RSPPTRVSIESITPKQRFPAPNFEQAYHSNIRYEQEESDNEEFENVVKNG
60 70 80 90 100
Pred CCCCCCCCCCCCCCHHHHCCCEECCCCCCCCCCCCCCCCCCCCCCCCCCC
AA HEASTNVFYESDGDDEEFVNEEYENSIDEESDDEGYSLNEDTTATNASFR
110 120 130 140 150
Pred CCCCCCCCCCCCCCCC
AA YPMNQRSTRKSQFYSSK
160
Legend:
Strand
Helix
Coil
Conf: + Confidence of prediction
Cart: 3-state assignment cartoon
Pred: 3-state prediction
AA: Target Sequence
B
Sad1-3V5 – wt 4A
Sad1-3V5
Tubulin
C
Sad1-GFP Sid4-mCherry DAPI mCherry/green
wt
sad1-4A
D
wt
sad1-1
sad1-4A
25° 32° 36°
E
Eic1/Mis19 -GFP Sid4-mCherry DAPI mCherry/green
wt
sad1-4A
F
Scm3-GFP Sid4-mCherry DAPI mCherry/green
wt
sad1-4A
G
Input α-Flag IP
Mis18-3Flag + – + + + – + +
Sad1-3V5 – wt wt 4A – wt wt 4A
Mis18-3Flag
Sad1-3V5
H
wt
csi1Δ
bub1Δ
sad1-4A
sad1-4A bub1Δ
sad1-4A bub1Δ
TBZ (µg/mL) 0 7.5 10 15
I
wt
sad1.1
sad1-4A
lem2Δ
sad1-4A lem2Δ
sad1-4A lem2Δ
TBZ (µg/mL) 0 7.5 12.5 15

Figure S5. The *sad1-4A* mutant disrupts localization of Mis18C components to SPBs and Mis18-Sad1 association, and exhibits specific genetic interactions. Related to Figure 5.

(A) PSIRPRED analysis of the Sad1-167 nucleoplasmic region identifies a helical domain covering residues 21-24.
(B) Western analysis comparing levels of Sad1-4A-3V5 and Sad1-3V5 protein expression using anti-V5 (α-V5) and anti-tubulin (Tat1).
(C) Sad1-GFP and mutant Sad1-4A-GFP protein localization with SPBs (Sid4-mCherry) in cells stained with DAPI. Scale bar: 5 μm.
(D) Serial dilution growth assays on YES media at 25, 32, and 36°C.
(E) Localization of Mis18C component Eic1/Mis19-GFP and SPB protein Sid4-mCherry in wild-type *sad1*$^{+}$ and mutant *sad1-4A* DAPI stained cells. Scale bar: 5 μm. Cells were grown, imaged, and quantified along with those in Figure 5B.
(F) Localization of Scm3-GFP and SPB protein Sid4-mCherry in wild-type *sad1*$^{+}$ and mutant *sad1-4A* DAPI stained cells. Scale bar: 5 μm.
(G) Western analyses of Input (extract) and anti-Flag Mis18-3Flag IPs to detect Sad1-3V5 (wt) or Sad1-4A-3V5 (4A) (anti-V5) and Mis18-3Flag (anti-Flag).
(H, I) Serial dilution growth assays of indicated strains on YES plates with or without TBZ added at indicated concentrations.

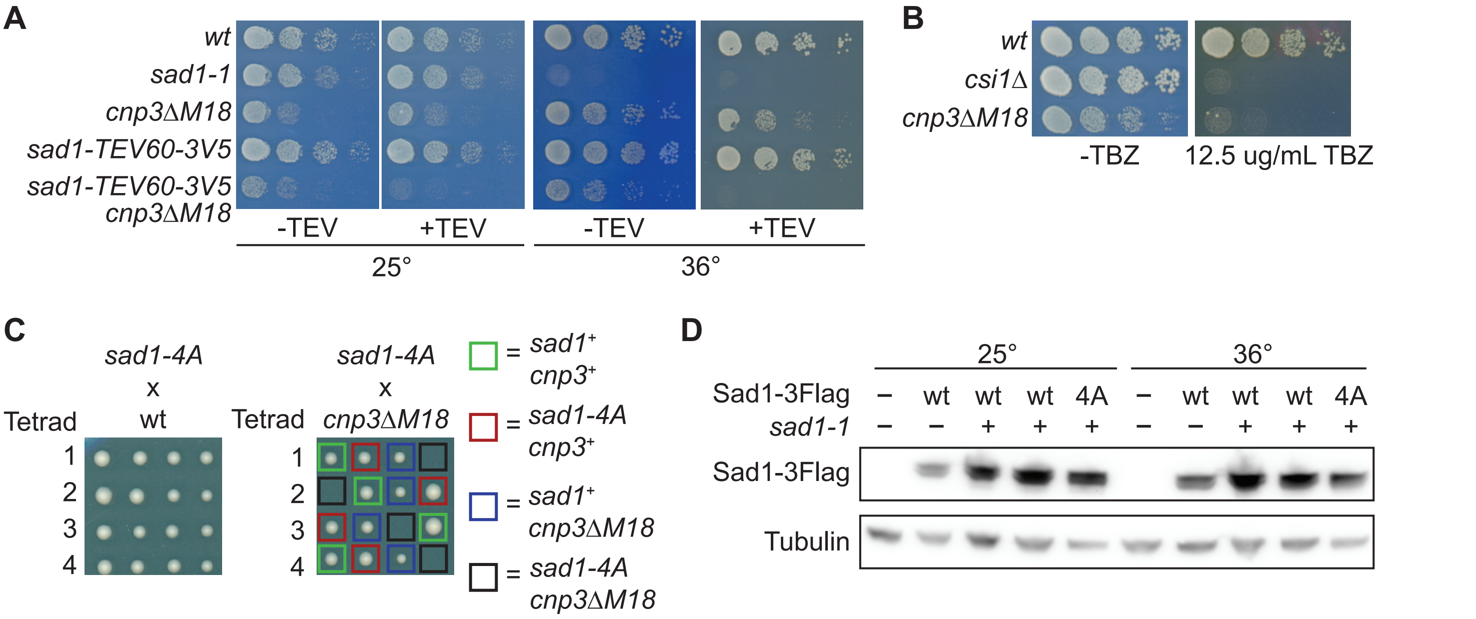

Figure S6. The *cnp3ΔM18* mutant is synthetic lethal with Sad1-TEV60-3V5 cleavage or the *sad1-4A* mutant. Related to Figure 6.

(A) Serial dilution growth assays of *cnp3ΔM18* mutant cells carrying *sad1-TEV60-3V5* with (+) or without (-) TEV expression on PMG plates at 25 or 36°C. wild-type (*wt*) and *sad1-1* cells provide controls.
(B) Serial dilution growth assays of wild-type (wt), *csi1Δ* and *cnp3ΔM18* cells on YES plates with or without TBZ.
(C) Growth of dissected spores from four tetrads resulting from crossing *sad1-4A* with wild-type (left) or *cnp3ΔM18* (right) cells. PCR genotyping confirmed that viable progeny did not carry both mutations as indicated by the colored boxes.
(D) Western (anti-Flag) to compare wild-type Sad1-3Flag (wt) and Sad1-4A-3Flag (4A) protein levels expressed from an ectopic locus (*e*) in cells with (+) or without (-) the *sad1-1* temperature sensitive mutation at the endogenous *sad1* locus. Loading control: anti-tubulin (Tat1).
